# A correlational study on moral distress and death attitude among oncology nurses in China

**DOI:** 10.3389/fpsyg.2025.1641964

**Published:** 2025-09-08

**Authors:** SiYing Xin, GuanMian Liang, QunFang Miao, YuTong Shao, JingYi Li

**Affiliations:** ^1^School of Nursing, Hangzhou Normal University, Hangzhou, Zhejiang, China; ^2^Nursing Department, Zhejiang Cancer Hospital, Hangzhou Institute of Medicine (HIM), Chinese Academy of Sciences, Hangzhou, Zhejiang, China; ^3^School of Clinical Medicine, Hangzhou Normal University, Hangzhou, Zhejiang, China; ^4^Nursing Department, The Second People's Hospital of Xiaoshan District, Hangzhou, Zhejiang, China

**Keywords:** oncology nurses, moral distress, death attitude, quantitative study, Chinese

## Abstract

**Background:**

Oncology nurses frequently care for terminally ill patients, which can expose them to moral stress and lead to moral distress. This dynamic negatively impacts their mental health and the quality of patient care. In Chinese society, where traditional cultural values often lead to an avoidance of the topic of death, nurses' attitudes toward death can be uniquely shaped, potentially affecting their ability to deliver effective end-of-life care. This study aimed to explore the prevalence of moral distress among Chinese oncology nurses and examine its relationship with attitudes toward death within this cultural context.

**Aim:**

To explore factors that influence moral distress among oncology nurses and their relationship with attitudes toward death.

**Methods:**

A cross-sectional study was conducted with oncology nurses (*n* = 264) from two hospitals in Zhejiang Province from February 2025 to April 2025, with participants selected by purposive sampling. Data were collected using a general demographic questionnaire, the moral distress scale (MDS), and the death attitude description scale (DAP-R).

**Results:**

The median MDS score was 72.00 (IQR: 48.25, 105.00), while the DAP-R yielded a median score of 97.00 (IQR: 89.00, 105.00). Significant negative correlations were found between individual responsibility, harm to the patient's interests, fear of death, death avoidance, approach acceptance, and escape acceptance (*P* < 0.05). Besides, failure to maintain the patient's maximum interests, value conflict, and death attitudes were significantly negatively correlated (*P* < 0.05). Regression analysis indicated that age, fear of death, and death avoidance were key factors influencing moral distress (*P* < 0.05).

**Conclusion:**

Our findings indicate that Chinese oncology nurses exhibit a moderate-to-high level of positive attitude toward death and a low level of moral distress. Besides, a negative correlation exists between moral distress and death attitudes. These findings suggest that Chinese cultural perspectives significantly influence nurses' attitudes toward death, which, in turn, shapes their moral decision-making and clinical practice. Nursing managers should consider implementing life-and-death education training to help nurses better understand death, strengthen their professional identity, and alleviate moral distress.

## 1 Introduction

In modern healthcare systems, oncology nurses operate in a high-pressure environment, facing numerous challenges such as staffing shortages, increasing complex patient care needs, and constantly evolving treatment guidelines (Özbaş et al., 2021; Shih et al., 2025). Previous research has indicated that such systems inherently generate moral stress during daily operations. This stress does not stem from a singular event but represents a chronic, structural ethical tension (Buchbinder et al., 2024). Nurses may encounter ethical dilemmas when they perceive that their efforts to mitigate patient distress are constrained by broader institutional or systemic barriers, thereby challenging their commitment to upholding high standards of care. However, this form of moral strain is not evenly distributed; instead, it frequently escalates during critical phases of patient care, manifesting as moral distress. Moral distress refers to a situation where an individual knows the morally right action but is unable to act in accordance with those moral principles due to various external constraints (Corley, 2002). The existing literature suggests that repeated exposure to moral distress can lead to emotions such as guilt, helplessness, self-doubt, and even depression, which adversely affect nurses' mental health (Zhao et al., 2024). In extreme cases, it may cause physical symptoms, including loss of appetite and sleep disturbances (Demir et al., 2024), and can contribute to occupational burnout, negatively impacting both the quality of care and job satisfaction (Yu et al., 2025). Oncology nursing is distinct from other specialties due to its continuous care and long treatment cycles, making it more likely for oncology nurses to form closer emotional bonds with patients, particularly those in advanced stages of cancer. In these cases, oncology nurses are required to provide emotional support and assist physicians with symptom management, which can further exacerbate the moral distress they experience.

Modern medical technology has prolonged the lives of terminally ill cancer patients through life-support interventions, yet the disease itself is seldom cured by these life-sustaining measures. Malignant tumors remain a prevalent cause of death, posing significant challenges for effective treatment. Accordingly, oncology nurses frequently face ethical dilemmas in their professional practice, which may lead to actions that conflict with established ethical standards. For instance, nurses may face ethical conflicts when navigating patient confidentiality and truth-telling about a diagnosis. Some patients desire full disclosure, while their family members may request that the nurse withhold this information (Pan et al., 2024). In such cases, nurses must balance the patient's right to know the truth with the family's desire to maintain confidentiality. Besides, cancer patients are often confronted with critical treatment decisions, such as choosing between invasive procedures or selecting an appropriate chemotherapy regimen. A lack of medical knowledge or fear of potential consequences can make it difficult for patients to make informed decisions, challenging nurses to ensure that patients are fully engaged in the decision-making process (Rabben et al., 2025). Moreover, when caring for patients in the terminal stages of cancer, families often insist on continuing treatment despite the patient's deteriorating quality of life, contributing to another source of moral distress for oncology nurses. In such cases, nurses are faced with the ethical conflict of balancing efforts to prolong survival with the need to improve the patient's quality of life (Ma et al., 2024). Previous studies have highlighted a strong correlation between the moral distress experienced by oncology nurses and their ability to care for terminally ill patients (Beiranvand et al., 2024; Corradi-Perini et al., 2021). Frequent exposure to moral distress not only negatively affects their psychological wellbeing but also compromises the quality of care they provide. Therefore, addressing the moral distress experienced by oncology nurses is crucial.

In Chinese culture, traditional concepts of family and filial piety (Lei et al., 2022) create an unavoidable responsibility for children to care for their parents in their final years. When confronted with terminally ill cancer patients, children often make significant efforts to prolong their parents' lives, driven by the desire to avoid familial criticism and prevent self-reproach after their parents' passing. However, such “overmedicalization” stands in direct contradiction to the principles of contemporary palliative care, resulting not only in unnecessary physical suffering for patients but also in the waste of medical resources, finances, and human capital. This cultural context further complicates the moral distress experienced by oncology nurses when caring for terminally ill cancer patients.

Attitudes toward death are an individual's evaluative and relatively stable emotional and psychological responses when confronted with death or terminally ill patients, often manifesting as fear, avoidance, or acceptance (Sun et al., 2019). Research has demonstrated that nurses' attitudes toward death significantly influence both their own occupational health and the quality of care they provide (Zhang S. et al., 2024). An individual's emotional experiences and behavioral responses are not only shaped by external events but are also regulated by their subjective cognition. Psychological and behavioral manifestations result from the complex cognitive processing of environmental stimuli (Al-Yagon et al., 2020). For oncology nurses, their attitudes toward death can, to some extent, influence their perception of death, which in turn could influence the emergence and management of ethical dilemmas.

Therefore, further investigation is warranted to determine whether oncology nurses' attitudes toward death influence their perception of patient death events and, subsequently, their levels of moral distress. While the majority of current research on moral distress emphasizes qualitative studies that explore respondents' authentic experiences (Deschenes et al., 2020), there is also a growing body of quantitative research examining the levels of moral distress among oncology nurses (Eche et al., 2023). However, existing quantitative studies on the relationship between moral distress and attitudes toward death remain limited, necessitating further inquiry to gain a deeper understanding of this connection. This study aimed to examine the current state of moral distress among oncology nurses in China and explore its correlation with their attitudes toward death. The ultimate goal is to provide theoretical foundations and practical recommendations for enhancing occupational health and improving nursing quality. By understanding the unique cultural influences in China, this research seeks to inform the development of tailored interventions that can improve the mental wellbeing of oncology nurses and enhance the quality of care they provide to terminally ill patients.

## 2 Method

### 2.1 Study design and sampling

This study employed purposive sampling and received ethical approval from the Ethics Committee of Hangzhou Normal University (No. 2024112). Inclusion criteria were as follows: (1) possession of a valid nurse practitioner license; (2) a minimum of 1 year of work experience in an oncology department; (3) provision of informed consent and voluntary participation. Exclusion criteria comprised: (1) nurses undergoing external training, internships, or further education within the hospital; (2) nurses currently on maternity, sick, or personal leave. Recruitment was conducted at one general hospital and one tertiary specialized hospital in Zhejiang Province, with data collection occurring from February to April 2025.

### 2.2 Participants

Based on the guideline that the sample size in multivariate analyses should be 5–10 times the number of independent variables (Ni et al., 2010), and accounting for a potential attrition rate of 10%−20%, the estimated required sample size ranged from 96 to 192. A total of 290 oncology nurses completed the survey. After excluding 26 invalid responses (those with uniform answers across all items), 264 valid questionnaires were retained, yielding an effective response rate of 91.03%. Of the participants, 14 were male (5.3%) and 250 were female (94.7%). The largest age group was 36–45 years, comprising 105 respondents (39.8%). Further demographic characteristics are detailed in Table 1.

**Table 1:** General demographic description statistics of respondents.

**Project**	**Category**	**Number**	**Constituent ratio (%)**
Gender	Male	14	5.3
	Female	250	94.7
Age	≤25 years old	57	21.6
	26–35 years old	81	30.7
	36–45 years old	105	39.8
	≥45 years old	21	8.0
Marital status	Married	153	58.0
	Unmarried	111	42.0
Years of working	≤5 years	89	33.7
	6–10 years	28	10.6
	11–15 years	64	24.2
	≥16 years	83	31.4
Education	College degree and below	7	2.7
	Undergraduate	233	88.3
	Master's degree or above	24	9.1
Title	Nurse	46	17.4
	Primary nurse	91	34.5
	Nurse-in-charge	101	38.3
	Co-chief superintendent nurse	22	8.3
	Chief superintendent nurse	4	1.5
Ever experienced the loss of a loved one	Yes	164	62.1
	No	100	37.9

### 2.3 Survey

The survey was administered to oncology nurses by trained researchers via an online Wenjuanxing platform. The questionnaire began with a standardized introduction explaining the study's purpose, significance, and instructions for completion, thereby ensuring informed consent. The researchers provided detailed instructions to ensure that participants understood the study's objectives and how to properly complete the questionnaire. Participants completed the questionnaire anonymously, with all items set as mandatory.

To ensure data integrity and minimize errors, each IP address was limited to a single submission, reducing the risk of duplicate or incomplete responses. The data collection procedure included real-time monitoring by the research team to ensure compliance and address any technical issues immediately. In addition, the research team reviewed the responses regularly to identify and correct any inconsistencies or outliers. Participation was entirely voluntary, and no incentives or compensation were provided to ensure impartiality.

To protect participants' mental wellbeing, a warning message was presented prior to any sensitive questions, informing them of their right to withdraw from the study at any time. In addition, a professional psychological support team was made available to assist participants who might experience distress. Access to psychological support was provided through platforms such as WeChat and DingTalk, ensuring participants could receive timely assistance if needed.

The questionnaire collected seven demographic variables: gender, age, marital status, years of working, educational level, title, and whether the participant had ever experienced the loss of a loved one.

### 2.4 Measures

***Moral Distress Scale for Nurses (MDS)***, developed by Corley (2002). Later, it was revised by Hamric and Blackhall (2007). In 2012, it was sinicized by Sun (2012). After cross-cultural adjustment, the Chinese version of MDS was formed. The scale consists of 22 items, including individual responsibility (8 items), failure to protect the patient's best interests (5 items), value conflict (6 items), and harm to the patient's interests (3 items). Each item measures both Moral Distress Frequency (MDF) and Moral Distress Intensity (MDI), both on a Likert scale of 5, with options ranging from “never/none” to “very frequent/severe” assigned a score of 0–4. The score of each item is the product of MDF and MDI, and the score ranges from 0 to 16 points. The scale's total score was the sum of the scores of all items, ranging from 0 to 352. The higher the score, the more serious the nurses' moral distress. The Cronbach coefficient for this scale in this study was 0.921.

***Death Attitude Profile-Revised (DAP-R)***, which was developed by Wong et al. (2015). In 1994, and then adapted by Tang et al. (2014) to form a Chinese version of DAP-R suitable for medical staff. The scale consists of fear of death (7 items), death avoidance (5 items), neutral acceptance (5 items), approach acceptance (10 items), and escape acceptance (5 items), with 32 items in five dimensions. Fear of death and death avoidance belong to a negative death attitude, while neutral acceptance, approach acceptance, and escape acceptance belong to a positive death attitude. Each item on the scale was scored on a Likert five-grade scale, and each item was scored from 1 to 5 points from “strongly disagree” to “strongly agree.” The death attitude tendency of the subjects was evaluated by evaluating the scores of each dimension. The Cronbach coefficient for this scale in this study was 0.863.

**Reliability and validity**. This study employed the Moral Distress Scale (MDS) and the Death Attitude Profile-Revised (DAP-R) as the primary instruments for data collection. Both tools have been extensively validated in previous research, demonstrating high internal consistency and strong construct validity. The MDS was designed to assess the moral distress experienced by healthcare professionals, while the DAP-R measures attitudes toward death. The MDS has a Cronbach's alpha coefficient of 0.879 (Sun, 2012), indicating strong reliability, and its construct validity has been supported through confirmatory factor analysis. The DAP-R has shown good psychometric properties, with a Cronbach's alpha of 0.875 (Tang et al., 2014), confirming its reliability and construct validity in similar populations.

### 2.5 Data analysis

EXCEL 2021 software was used to establish the database, and SPSS 27.0 software was used for statistical analysis. Non-normally distributed measurement data were described using median and quartiles, while count data were described using frequency, percentage, or percentage. Chi-square analysis was used to analyze the differences in the moral distress of nurses with different demographic characteristics. The Spearman correlation method was used to investigate the relationship between death attitude and the moral distress of nurses in the oncology department. Multiple linear regression was used to analyze the influence of death attitude and demographic data on the moral distress of nurses in the oncology department. For reliability testing, a Cronbach's alpha coefficient > 0.7 (Tavakol and Dennick, 2011) demonstrated good internal consistency. Statistical significance was set at *p* < 0.05.

### 2.6 Ethics considerations

This study was approved by the Ethics Committee of Hangzhou Normal University, which approved our study (No. 2024112). During the study, the researchers adhered to the principles of informed consent, ensuring that all participants voluntarily agreed to participate and signed consent forms before data collection. Additionally, the study followed strict confidentiality protocols. All survey results were used solely for academic research purposes, and personal information was anonymized by using numerical codes in place of identifying details during data entry to ensure participant privacy.

## 3 Results

### 3.1 Oncology nurses' death attitude and moral distress scale score

The median total score for oncology nurses' attitudes toward death was 97.00 (IQR: 89.00, 105.00), with a median average item score of 3.03 (IQR: 2.78, 3.28). The negative death attitude subscale had a total score of 37.00 (IQR: 33.00, 41.00), corresponding to an average item score of 3.08 (IQR: 2.75, 3.42). The positive death attitude subscale yielded a median total score of 60.00 (IQR: 54.00, 65.00), with a median average item score of 3.00 (IQR: 2.70, 3.25). Among the various dimensions of death attitude, the median average scores ranked from highest to lowest were: fear of death, 3.29 (IQR: 3.00, 3.71); neutral acceptance, 3.20 (IQR: 2.80, 3.40); approach acceptance, 3.00 (IQR: 2.60, 3.20); escape acceptance 3.00 (IQR: 2.60, 3.40); and death avoidance 2.80 (IQR: 2.40, 3.20).

The median total score for moral distress was 72.00 (IQR: 48.25, 105.00), with a median average item score of 3.27 (IQR: 2.19, 4.77). Dimension-wise, the median average item scores ranked from highest to lowest were as follows: personal responsibility, 4.00 (IQR: 2.63, 5.50), failure to protect the patient's best interests, 3.00 (IQR: 1.40, 4.60), harm to the patient's interests, 3.00 (IQR: 1.33, 4.33), and value conflict, 2.50 (IQR: 1.33, 4.33). Detailed dimension scores are presented in Table 2.

**Table 2:** Total scores and dimension scores of death attitude and moral distress scale.

**Dimensions**	**Score**	**Average score of entries**
**Total score of attitude toward death**	97.00 (89.00, 105.00)	3.03 (2.78, 3.28)
Negative attitude toward death	37.00 (33.00, 41.00)	3.08 (2.75, 3.42)
Fear of death	23.00 (21.00, 26.00)	3.29 (3.00, 3.71)
Death avoidance	14.00 (12.00, 16.00)	2.80 (2.40, 3.20)
Positive attitude toward death	60.00 (54.00, 65.00)	3.00 (2.70, 3.25)
Neutral acceptance	16.00 (14.00, 17.00)	3.20 (2.80, 3.40)
Approach acceptance	30.00 (26.00, 32.00)	3.00 (2.60, 3.20)
Escape acceptance	15.00 (13.00, 17.00)	3.00 (2.60, 3.40)
**Total score of moral distress**	72.00 (48.25, 105.00)	3.27 (2.19, 4.77)
Individual responsibility	32.00 (21.00, 44.00)	4.00 (2.63, 5.50)
Failure to protect the patient's best interests	15.00 (7.00, 23.00)	3.00 (1.40, 4.60)
Value conflict	15.00 (8.00, 26.00)	2.50 (1.33, 4.33)
Harm to the patient's interests	9.00 (4.00, 13.00)	3.00 (1.33, 4.33)

### 3.2 Analysis of differences in the moral distress of demographic data of oncology nurses

The results showed that age, marital status, working years, and professional title were significantly associated with moral distress (*P* < 0.05). Other demographic variables showed no significant association with moral distress (*P* > 0.05), as shown in Table 3.

**Table 3:** Analysis of differences in moral distress among different demographic groups.

**Project**	**Category**	**Number**	**Moral distress**	**χ^2^**	***P***
Gender	Male	14	75.00 (28.50, 121.50)	131.909	0.388
	Female	250	72.00 (48.75, 138.70)		
Age	≤25 years old	57	136.00 (121.00, 158.00)	698.275	< 0.001
	26–35 years old	81	91.00 (82.50, 100.00)		
	36–45 years old	105	50.00 (33.00, 57.00)		
	≥45 years old	21	16.00 (13.00, 19.00)		
Marital status	Married	153	52.00 (30.50, 65.00)	197.039	< 0.001
	Unmarried	111	111.00 (88.00, 138.00)		
Years of working	≤5 years	89	120.00 (98.00, 143.50)	451.646	0.010
	6–10 years	28	89.50 (81.00, 98.50)		
	11–15 years	64	62.00 (49.00, 84.00)		
	≥16 years	83	40.00 (22.00, 53.00)		
Education	College degree and below	7	103.00 (50.00, 151.00)	259.584	0.426
	Undergraduate	233	71.00 (47.00, 105.00)		
	Master's degree or above	24	78.00 (53.75, 106.00)		
Title	Nurse	46	129.00 (113.75, 153.25)	622.185	< 0.001
	Primary nurse	91	93.00 (77.00, 107.00)		
	Nurse-in-charge	101	52.00 (34.00, 60.50)		
	Co-chief superintendent nurse	22	29.00 (15.75, 48.50)		
	Chief superintendent nurse	4	27.50 (13.75, 56.25)		
Ever experienced the loss of a loved one	Yes	164	71.00 (45.00, 102.00)	123.687	0.591
	No	100	85.50 (50.00, 112.50)		

P-value: The p-value indicates whether the observed relationship is statistically significant. A p-value < 0.05 is considered statistically significant, meaning that this relationship is unlikely to have occurred by chance.

### 3.3 Correlation analysis between death attitude and moral distress of oncology nurses

Analysis of the correlation between death attitude and moral distress found that the individual responsibility dimension of moral distress exhibited a significant negative correlation with fear of death, death avoidance, approach acceptance, and escape acceptance (all *P* < 0.05). Similarly, failure to protect the patient's best interests and value conflict were significantly negatively correlated with fear of death, death avoidance, neutral acceptance, approach acceptance, and escape acceptance (all *P* < 0.05). Besides, damage to the patient's interests was negatively correlated with fear of death, death avoidance, approach acceptance, and escape acceptance (all *P* < 0.05), as shown in Table 4.

**Table 4:** Correlation analysis between death attitude dimensions and moral distress dimensions.

**project**	**Individual responsibility**	**Failure to protect the patient's best interests**	**Value conflict**	**Harm to the patient's interests**	**Total score of moral distress**
Fear of death	−0.362^*^	−0.331^*^	−0.333^*^	−0.313^*^	–
Death avoidance	−0.341^*^	−0.379^*^	−0.285^*^	−0.256^*^	–
Neutral acceptance	−0.118	−0.167^*^	−0.138^*^	−0.098	–
Approach acceptance	−0.341^*^	−0.289^*^	−0.294^*^	−0.291^*^	–
Escape acceptance	−0.144^*^	−0.133^*^	−0.201^*^	−0.133^*^	–
Total score of attitude toward death	–	–	–	–	−0.428

* *p* < 0.05; “–” means no data.

P-value: The p-value indicates whether the observed relationship is statistically significant. A p-value < 0.05 is considered statistically significant, meaning that this relationship is unlikely to have occurred by chance.

### 3.4 Multivariate linear regression analysis of nurses' moral distress in the oncology department

Multivariate linear regression analysis was performed using the variables with statistical significance and no collinearity from the univariate analysis as independent variables. The total score from the Oncology Nurses' Moral Distress Scale served as the dependent variable. See Table 5 for the independent variable assignment method.

**Table 5:** Independent variable assignment.

**Independent variable**	**Assignment**
Age	≤25 years old = 1, 26–35 years old = 2, 36–45 years old = 3, ≥46 years old = 4
Marital status	Married = 1, Unmarried = 2
Years of working	≤5 years = 1, 6–10 years = 2, 11–15 years = 3, ≥16 years = 4
Title	Nurse = 1, Primary nurse = 2, nurse-in-charge = 3, co-chief superintendent nurse = 4, chief superintendent nurse = 5
Fear of death	Original value entry
Death avoidance	Original value entry
Neutral acceptance	Original value entry
Approach acceptance	Original value entry
Escape acceptance	Original value entry

The multicollinearity test showed that all included variables had variance inflation factor values < 10.0, confirming the absence of multicollinearity between variables. The Durbin-Watson test yielded a value of 1.297, which fell within the acceptable range of 0–4, indicating data independence. Besides, the residual analysis showed that the residuals conformed to a normal distribution. The multiple linear regression analysis results showed that marital status, fear of death, and death avoidance were significant predictors of moral distress after their incorporation into the regression equation (*P* < 0.05), accounting for 86.9% of the total variance. This finding indicated that age, fear of death, and death avoidance were key factors influencing moral distress among oncology nurses. See Table 6 for details.

**Table 6:** Multiple linear regression analysis of death attitude dimensions on moral distress.

**Variable**	***B***	**SE**	**β**	***t***	***P***	**VIF**
Constant term	242.067	11.978	–	20.210	< 0.001	–
Age	−40.315	2.649	−0.791	−15.217	< 0.001	5.442
Marital status	6.558	3.732	0.070	1.757	0.080	3.227
Years of working	1.184	2.043	0.032	0.580	0.563	6.156
Title	−0.006	2.131	0.000	−0.003	0.998	3.670
Fear of death	−1.916	0.300	−0.166	−6.392	< 0.001	1.354
Death avoidance	−2.518	0.415	−0.178	−6.068	< 0.001	1.734
Neutral acceptance	0.885	0.480	0.054	1.844	0.066	1.699
Approach acceptance	−0.311	0.264	−0.041	−1.180	0.239	2.449
Escape acceptance	−0.436	0.438	−0.033	−0.996	0.320	2.273

R = 0.935, R^2^ = 0.874, adjusted R^2^ = 0.869, F = 195.340, P < 0.001, “–” means no data.

P-value: The p-value indicates whether the observed relationship is statistically significant. A p-value < 0.05 is considered statistically significant, meaning that this relationship is unlikely to have occurred by chance.

## 4 Discusses

### 4.1 Death attitudes and moral distress among the oncology nurses

The results of this study indicate that oncology nurses' attitudes toward death are primarily characterized by fear and avoidance, particularly among younger nurses. The median total score for attitudes toward death was 97.00, with negative attitudes being more prevalent than positive ones. The finding that fear of death is a dominant element, especially among younger nurses, is consistent with previous research and reflects that younger nurses have limited experience in end-of-life care in clinical practice, which can heighten their fear of death (Maestro-González et al., 2025).

Our results further suggest that oncology nurses' attitudes toward death are influenced not only by clinical experience and age but also by cultural factors. The role of cultural background in shaping attitudes toward death cannot be overlooked. Traditional Chinese culture is often characterized by an optimistic, life-oriented perspective, emphasizing the value of living while frequently avoiding the contemplation or discussion of death. Traditional death taboos influence many Chinese individuals, leading to a reluctance to engage with the topic, often advocating the concept of “better dead than alive” (Liu Y. et al., 2023). In Chinese traditional culture, death is often considered a taboo subject, and many nurses, due to cultural practices and psychological avoidance, tend to avoid discussing death-related matters when confronted with it (Tu et al., 2022). This cultural factor contributes to nurses' lack of effective coping strategies and psychological preparedness for death, especially when caring for terminally ill patients, thus intensifying their fear of death and moral distress.

This study also found that the level of moral distress among oncology nurses was lower than that reported in previous studies involving ICU nurses (Yuan et al., 2023). One possible explanation for this difference is the predictable and progressive nature of cancer. This predictability helps alleviate caregivers' grief and reduces moral conflicts (Huang et al., 2023). Furthermore, terminal cancer patients are less likely to undergo invasive life-sustaining treatments, which reduces the ethical burden on nurses to make complex ethical decisions, such as whether to continue or discontinue treatment, thereby lowering the level of moral distress (Liu C. et al., 2023).

Among the various dimensions assessed, the sense of individual responsibility received the highest score, indicating that nurses generally feel a strong sense of duty when caring for patients. A moderate level of responsibility has been shown to improve the quality of care and enhance nurses' empathy (Hao et al., 2019). However, high levels of empathy may lead to feelings of helplessness and frustration, as nurses may mistakenly believe they can alleviate patients' suffering. This belief can result in a sense of futility when witnessing the deterioration and death of patients (Arimon-Pagès et al., 2019). Therefore, it is crucial to help nurses establish realistic psychological expectations in clinical practice to mitigate the negative impact of excessive responsibility and empathy.

### 4.2 Influencing factors of moral distress among the oncology nurses

#### 4.2.1 Age

Age emerged as a significant negative predictor of moral distress (β = −0.791, *p* < 0.001), with nurses aged ≥45 reporting the lowest moral distress scores, and those ≤ 25 years old reporting the highest. Interestingly, neither years of experience nor professional title significantly influenced moral distress levels. The lower distress levels in older nurses may be attributed to greater exposure to life-and-death situations, fostering greater psychological maturity and acceptance of death. This is consistent with prior findings demonstrating that emotional regulation training can enhance nurses' death attitudes and alleviate ethical distress (Szekely and Miu, 2015), offering practical insights for targeted interventions among younger nurses.

#### 4.2.2 Fear of death

Fear of death was also identified as a significant negative predictor (β = −0.166, *p* < 0.001) of moral distress scores. Accordingly, nurses with heightened death anxiety may cope by avoiding end-of-life decision-making or by emotionally detaching themselves from patients to self-protect, thereby reducing their perception of ethical responsibility. This aligned with research suggesting that individuals often adopt defensive avoidance behaviors following traumatic experiences (Rompilla et al., 2022). An intense fear of mortality not only compromises care quality but may also increase the likelihood of turnover intention among nursing staff (Chang and Lin, 2023). While temporary avoidance may serve as a coping mechanism to mitigate the psychological toll of patient loss, sustained avoidance risks undermining their professional accountability and hindering sustained career and personal growth.

#### 4.2.3 Death avoidance

Death avoidance (β = −0.178, *p* < 0.001) also emerged as a significant negative predictor of moral distress. Nurses who actively avoid death-related situations may engage in less ethical reflection, thereby experiencing less distress over issues like patient rights. Prior research highlights that, although healthcare professionals generally support the principle of delivering bad news, relatively few do so in practice (Sun et al., 2023). In China's traditionally physician-dominated healthcare system, hierarchical power structures often limit nurses' involvement in decision-making, exacerbating moral alienation (Sun et al., 2022).

#### 4.2.4 Other factors

Neutral acceptance of death demonstrated a weak but positive association with moral distress (β = 0.054, *p* = 0.066), suggesting that even a positive outlook toward death does not preclude moral conflicts, especially in contexts involving patient-clinician disagreements. Similarly, being unmarried showed a marginal positive association with moral distress (β = 0.070, *p* = 0.080), possibly due to a lack of emotional support. Family support has been shown to significantly affect nurses' psychological wellbeing, with higher perceived familial support correlating with stronger moral value orientations (Lok et al., 2023). These findings underscore the importance of organizational support structures, such as peer mentoring and emotional support systems, particularly for younger or unmarried nurses. Hospital administrators could implement tiered interventions, e.g., cognitive training, mentorship from senior nurses, and policy reforms, to clarify nurses' ethical roles, reconcile short-term coping strategies with long-term moral development, and ultimately reduce moral distress.

### 4.3 Intervention strategy establishment

The results of this study suggest a close relationship between death attitudes and moral distress among oncology nurses, indicating that the emotions and attitudes nurses experience when facing patient death may significantly influence the occurrence of moral distress. Therefore, hospitals should strengthen death education and coping skills training for oncology nurses, making it an integral part of their work and daily practice. Indeed, Western models of death education are not entirely applicable to traditional Chinese culture (Zhang H. et al., 2024). Western death education typically emphasizes individual autonomy and personal rights, while China places greater emphasis on collectivism and family responsibility (Gao et al., 2021). This results in Chinese nurses often needing to find a balance between the wishes of family members and patients when facing death. Therefore, when designing death education training, these cultural differences should be taken into account. Integrating the concept of “filial piety” from Confucianism (Lei, 2025) could help meet the cultural and psychological needs of Chinese nurses and enable them to better manage communication and emotional support with patients and families. This approach would not only reduce moral distress but also improve the cultural acceptance and effectiveness of death education.

In practice, death education should not be limited to theoretical teaching but should also include flexible, practical applications. For instance, cognitive restructuring strategies have been shown to effectively reduce the negative impact of loss events (Elinger et al., 2023). In this context, nursing managers should guide oncology nurses to develop a deep understanding of the patient's condition, help them set realistic care goals, and learn to rationally accept the limitations of medical technology, thereby reducing the occurrence of moral distress. This process should also involve comprehensive communication with patients and their families, clarifying treatment goals, and respecting the patient's wishes (Zhu et al., 2023).

To further alleviate moral distress, nursing managers can assist oncology nurses in aligning treatment goals and patient preferences through Advance Care Planning (ACP) training. The practice of ACP communication helps healthcare providers serve as a bridge between patients and families, clarifying end-of-life wishes and reducing negative emotions at the time of death (Wang et al., 2019). In this process, the nurse's role extends beyond technical communication to include emotional support and psychological counseling, thereby reducing the moral burden on all healthcare providers.

## 5 Strengths and limitations

This study systematically examines the factors influencing moral distress among oncology nurses in China and explores the relationship between moral distress and nurses' attitudes toward death. By incorporating the distinct cultural context of the Chinese healthcare system, this research addresses a gap in the literature regarding non-Western cultural perspectives in this area. The findings highlight the significant impact of cultural factors on nurses' perceptions of death and offer context-specific intervention recommendations.

A limitation of this study is its focus on a single city within Zhejiang Province. Future research should broaden the sample scope to mitigate geographical biases and enhance the precision of study outcomes.

## 6 Conclusions

Importantly, this study identified a correlation between moral distress among oncology nurses and their age, as well as two negative attitudes toward death: fear of death and avoidance of death. To address these issues, nursing managers implement a comprehensive death education and training program for oncology nurses that integrates both scientific and humanistic approaches to death. This program should be culturally adapted to align with Chinese norms by incorporating values such as family responsibility and “filial piety.” Besides, the training should include practical applications, such as cognitive restructuring strategies, to mitigate the impact of loss, guide nurses in setting realistic care goals, and improve communication with patients and families to honor patients' wishes. Furthermore, nursing managers should establish a comprehensive mental health support system to alleviate moral distress, with emphasis on expanding nurses' roles to include emotional support and psychological counseling. By enhancing nurses' understanding of death and strengthening their psychological resilience, hospitals can help oncology nurses cultivate a more rational and balanced perspective on death, thereby improving both their wellbeing and the quality of care they provide.

## Data Availability

The raw data supporting the conclusions of this article will be made available by the authors, without undue reservation.
